# Symptom Duration and Resolution With Early Outpatient Treatment of Convalescent Plasma for Coronavirus Disease 2019: A Randomized Trial

**DOI:** 10.1093/infdis/jiad023

**Published:** 2023-01-31

**Authors:** Sheriza N Baksh, Sonya L Heath, Yuriko Fukuta, David Shade, Barry Meisenberg, Evan M Bloch, Aaron A R Tobian, Emily S Spivak, Bela Patel, Jonathan Gerber, Jay S Raval, Donald Forthal, James Paxton, Giselle Mosnaim, Shweta Anjan, Janis Blair, Edward Cachay, Judith Currier, Piyali Das, Moises Huaman, Catherine Sutcliffe, Anusha Yarava, Arturo Casadevall, David Sullivan, Daniel Hanley, Kelly A Gebo

**Affiliations:** Department of Epidemiology Bloomberg Johns Hopkins School of Public Health, Baltimore, Maryland, USA; Department of Medicine, Division of Infectious Diseases, University of Alabama at Birmingham, Birmingham, Alabama, USA; Department of Medicine, Section of Infectious Diseases, Baylor College of Medicine, Houston, Texas, USA; Department of Epidemiology Bloomberg Johns Hopkins School of Public Health, Baltimore, Maryland, USA; Department of Medicine and Research Institute of Luminis Health, Annapolis, Maryland, USA; Department of Pathology, Johns Hopkins University School of Medicine, Baltimore, Maryland, USA; Department of Pathology, Johns Hopkins University School of Medicine, Baltimore, Maryland, USA; Department of Medicine, Division of Infectious Diseases, University of Utah, Salt Lake City, Utah, USA; Department of Medicine, Divisions of Pulmonary and Critical Care Medicine, University of Texas Health Science Center, Houston, Texas, USA; Department of Medicine, Division of Hematology and Oncology, University of Massachusetts, Worchester, Massachusetts, USA; Department of Pathology, University of New Mexico, Albuquerque, New Mexico, USA; Department of Medicine, Division of Infectious Diseases, University of California, Irvine, Irvine, California, USA; Department of Emergency Medicine, Wayne State University, Detroit, Michigan, USA; Department of Medicine Northshore University Health System, Division of Allergy and Immunology, Evanston, Illinois, USA; Department of Medicine, Division of Infectious Diseases, University of Miami, Miami, Florida, USA; Department of Medicine, Division of Infectious Diseases, Mayo Clinic, Phoenix, Phoenix, Arizona, USA; Department of Medicine, Division of Infectious Diseases, University of California, San Diego, La Jolla, California, USA; Department of Medicine, Division of Infectious Diseases, University of California, Los Angeles, Los Angeles, California, USA; Department of Neurology, Brain Injury Outcomes Division, Johns Hopkins University School of Medicine, Baltimore, Maryland, USA; Department of Medicine, Division of Infectious Diseases, University of Cincinnati Medical Center, Cincinnati, Ohio, USA; Department of Epidemiology Bloomberg Johns Hopkins School of Public Health, Baltimore, Maryland, USA; Department of Neurology, Brain Injury Outcomes Division, Johns Hopkins University School of Medicine, Baltimore, Maryland, USA; Departments of Molecular Microbiology and Immunology, Johns Hopkins Bloomberg School of Public Health, Baltimore, Maryland, USA; Departments of Molecular Microbiology and Immunology, Johns Hopkins Bloomberg School of Public Health, Baltimore, Maryland, USA; Department of Neurology, Brain Injury Outcomes Division, Johns Hopkins University School of Medicine, Baltimore, Maryland, USA; Department of Medicine, Division of Infectious Diseases, Johns Hopkins University School of Medicine, Baltimore, Maryland, USA

**Keywords:** COVID-19, COVID-19 serotherapy, plasma, symptom duration

## Abstract

**Background:**

Coronavirus disease 2019 (COVID-19) convalescent plasma (CCP) reduces hospitalizations among outpatients treated early after symptom onset. It is unknown whether CCP reduces time to symptom resolution among outpatients.

**Methods:**

We evaluated symptom resolution at day 14 by trial arm using an adjusted subdistribution hazard model, with hospitalization as a competing risk. We also assessed the prevalence of symptom clusters at day 14 between treatments. Clusters were defined based on biologic clustering, impact on ability to work, and an algorithm.

**Results:**

Among 1070 outpatients followed up after transfusion, 381 of 538 (70.8%) receiving CCP and 381 of 532 (71.6%) receiving control plasma were still symptomatic (*P* = .78) at day 14. Associations between CCP and symptom resolution by day 14 did not differ significantly from those in controls after adjustment for baseline characteristics (adjusted subdistribution hazard ratio, 0.99; *P* = .62). The most common cluster consisted of cough, fatigue, shortness of breath, and headache and was found in 308 (57.2%) and 325 (61.1%) of CCP and control plasma recipients, respectively (*P* = .16).

**Conclusions:**

In this trial of outpatients with early COVID-19, CCP was not associated with faster resolution of symptoms compared with control. Overall, there were no differences by treatment in the prevalence of each symptom or symptom clusters at day 14.

**Clinical Trials Registration:**

NCT04373460.

## Teaser

Early treatment with coronavirus disease 2019 convalescent plasma was not associated with faster symptom resolution than control treatment, in contrast with decreased hospitalization in the same population, suggesting that the timing antibody treatment may affect hospitalization and symptom progression differently.

With the introduction of the novel severe acute respiratory syndrome coronavirus 2 (SARS-CoV-2) virus in early 2020, clinicians encountered atypical symptom profiles [1] and a wide range of symptom duration [2], unlike the pathologic mechanism of known viral respiratory pathogens, which usually resolved within 2 weeks of symptom onset [3]. As the SARS-CoV-2 epidemic grew to become a pandemic, the case definition evolved [4], as did our collective understanding of the impact on quality of life and the ability to return to work [5].

Previous studies have shown variability in the time to resolution of symptoms after acute coronavirus disease 2019 (COVID-19) infection [6–8]. A July 2020 Centers for Disease Control and Prevention report of COVID-19 symptom duration noted that 35% of 270 participants experienced ill health 2–3 weeks after symptom onset [9]. Another study from Israel found that most patients reported symptoms lasting 2 weeks, with anosmia and fatigue lasting 3–4 weeks [10]. In addition, investigators noted a persistent cluster of fatigue, breathing difficulty and ageusia/anosmia 6 months after illness onset.

From January to May 2020, a study of approximately 3000 confirmed COVID-19 cases reported 60% of participants with improvement at 20 days, 80% at 30 days, and 91% at 60 days [11]. Given the variable characterization of COVID-19 symptoms, studies of treatments to reduce symptom duration and severity are needed, particularly with respect to symptoms inhibiting one's ability to work. Factors associated with prolonged recovery from SARS-CoV-2 include older age, underlying comorbid conditions, female sex, severity of illness, higher body mass index (BMI), diabetes mellitus, lack of vaccination, and SARS CoV-2 variant [9, 12, 13]. However, the impact of early treatment on resolution of acute COVID-19 symptoms in the acute phase of illness (within 14 days) has not been well studied among outpatients receiving antibody therapy.

We investigated the influence of COVID-19 convalescent plasma (CCP) versus control plasma on resolution of symptoms from the day of transfusion through day 14 of follow-up as part of a randomized controlled trial [14]. This analysis explores the resolution of all symptoms, the resolution of symptoms closely aligned with the ability to return to work, and the clustering of symptoms at day 14. We also explored the change in severity of symptoms by day 14 between treatments for 4 symptoms with higher impact on overall day-to-day functioning: cough, shortness of breath, fatigue, and headache. We chose these symptoms for an in-depth look at changes in severity owing to their impact on quality of life, including at work.

## METHODS

### Study Population

The Convalescent Plasma to Limit SARS-CoV-2 Associated Complications (CSSC-004) trial was a double-masked, multicenter, randomized, controlled trial investigating the use of CCP to prevent hospitalization and death among outpatients, compared with control plasma [14]. The trial recruited 1225 symptomatic, SARS-CoV-2–positive adults from 3 June 2020 to 1 October 2021. Of these, 1181 participants received a transfusion of either CCP or control plasma, standard SARS-CoV-2 nonimmune plasma samples collected before 1 January 2020 or seronegative for SARS-CoV-2. All trial participants were transfused within 9 days after symptom onset. For this report, we restricted analysis to the 1070 participants with complete symptom data on the day of transfusion (day 0) through day 14 of follow-up.

### Institutional Review Board Approvals

All study activities were approved by the Johns Hopkins University single institutional review board, the Navajo Nation Human Research Review Board, the Indian Health Service National Institutional Review Board, and the Human Research Protection Office of the US Department of Defense. All study activities were in accordance with the Declaration of Helsinki, Good Clinical Practice guidelines of the International Conference on Harmonization, and all applicable regulatory requirements. Written informed consent was obtained from all study participants.

### Outcomes

The primary outcome for this study, prespecified in the statistical analysis plan for CSSC-004, was the difference in time to resolution of all symptoms by day 14, defined as the absence of all queried symptoms by day 14 of follow-up, between the CCP and control plasma treatment groups. Participants were censored at the time of symptom resolution, loss to follow-up, or death. The CSSC-004 trial participants were asked about the presence and severity of symptoms at days 0, 1, 3, 5, 7, 10, and 14 through a structured, self-report form administered via phone (days 1, 3, 5, 7, and 10) and in-person visits (days 0 and 14). Data were collected on self-reported cough, fatigue, shortness of breath, ageusia, anosmia, nasal congestion, headache, myalgia, neurologic changes (ie, changes in cognitive functioning), nausea/vomiting, sore throat, diarrhea, skin manifestations (ie, acral lesions, rash, or swelling), chills, and fever at each visit. Details on symptom severity can be found in Supplementary Table 1.

We also examined the difference between treatment groups in the proportion of participants experiencing a symptom within specified symptom clusters at day 14. We took 4 approaches to define symptom clusters before analysis of day 14 data. Clusters 1 (any of cough, fatigue, shortness of breath, or headache) and 2 (any neurologic symptoms, loss of taste, or loss of smell) were defined based on common clinical presentations of symptoms. Cluster 3 was investigator defined, based on a symptom's potential to inhibit an individual's ability to return to work after acute illness. For cluster 3, we adjusted the severity levels for assessing the presence of symptoms before analysis in order to develop a clinically meaningful definition for symptom resolution. Specifically, cluster 3 consisted of any fever, fatigue (grade 2+), diarrhea, headache (grade 2+), myalgia (grade 2+), or vomiting.

In addition, we took an unsupervised, machine learning, algorithmic approach to derive data-driven symptom clusters, based on the prevalence of each symptom at each visit in the CSSC-004 study population [15]. This analysis allowed us to prespecify the number of clusters to minimize intracluster variation. Using k-means clustering, we allowed for the use of 2, 3, or 4 clusters and chose the optimal number of clusters with the lowest intracluster variation using the silhouette method. We then assessed the proportion of participants with symptoms within all clusters at day 14.

The presence of a symptom cluster was determined when a participant experienced ≥1 of the component symptoms within a cluster. For example, a participant reporting grade 2 fatigue and grade 1 cough at day 14 would be counted as having cluster 1 (cough or fatigue) and cluster 3 (fatigue at grade 2 or higher) symptoms at day 14. Finally, we explored differences between treatment groups in the change in severity for cluster 1 symptoms between treatment groups.

### Variable Definition

Age was recorded at the time of transfusion. Sex (male or female), race (white, black, other, or multirace) and ethnicity (Hispanic or non-Hispanic) were self-reported at enrollment. BMI was calculated using height and weight at enrollment. The date of transfusion was used as a surrogate for variant. Transfusions before 15 June 2021 were classified as during the pre-Alpha/Alpha wave, and those from 15 June to 1 October 2021 as during the Delta wave. We used the self-reported time of symptom onset to calculate the time from symptom onset to transfusion. Baseline comorbid conditions were self-reported at day 0. Vaccination status was defined as fully vaccinated (≥2 weeks from the final dose in the primary vaccine series to transfusion), partially vaccinated (receipt of 1 dose in a 2-dose primary vaccine series or <2 weeks from the final dose in the primary vaccine series to transfusion) or unvaccinated.

### Statistical Analysis

We used χ^2^ statistics for categorical covariates and the Wilcoxon rank sum test for continuous covariates to compare differences at baseline between patients transfused with CCP and those transfused with control plasma. We then used χ^2^ statistics to assess differences between treatment groups in symptoms and symptom clusters at day 14. To assess differences in the change in severity for cough, shortness of breath, fatigue, and headache, we used a *t* test for the distribution of change scores at day 14. We used a competing risk survival analysis to evaluate the association between transfusion of CCP and resolution of symptoms, with competing risks of death or hospitalization.

Crude and adjusted models were used to compare the subdistribution hazards of symptom resolution between treatment groups. In the adjusted model, we adjusted for age, sex, race, BMI, diabetes mellitus, time from symptom onset to transfusion, vaccination status, baseline C-reactive protein level, and variant wave. These covariates were specified before analysis, based on clinical evidence suggesting an association with symptoms. We conducted subgroup analyses for the time to resolution of symptoms between treatment groups for sex, age, BMI, race, ethnicity, diabetes mellitus, time from symptom onset to transfusion, and variant wave. Using an unadjusted ordinal logistic model, we assessed differences in the change in severity of cluster 1 symptoms for a subgroup of those reporting the highest severity of those symptoms at baseline. All analyses were conducted using R software, version 4.0.3.

To examine the robustness of our results, we first assessed the sensitivity of our results to participants whose symptoms resolved before day 3. For this, we did not count resolution of symptoms before day 3 as an event; however, these participants contributed follow-up time to the analysis and were censored at the time of resolution. We then assessed the sensitivity of our results to the inclusion of fully vaccinated individuals by excluding those who were fully vaccinated from the analysis. Finally, we assessed the sensitivity of our results to the inclusion of those who were hospitalized (n = 54) during follow-up by excluding them from the analysis.

## RESULTS

### Population Characteristics

Of the 1070 participants in CSSC-004 with complete symptom data through day 14 who were randomized and transfused with either CCP (n = 538) or control plasma (n = 532), 291 (54.1%) in the CCP and 318 (59.8%) in the control group were female (*P* = .07). The median age (interquartile range [IQR]) was 43.0 (33–54) years in the CCP group and 44.5 (33–55) years in the control group, (*P* = .38). Regarding race, 422 (78.4%) in the CCP and 436 (82.0%) in the control group were white (*P* = .17), and 85 (12.1%) and 77 (14.5%), respectively, were Hispanic/Latino (*P* = .29). Clinical characteristics in the CCP and control groups were similar at baseline. Approximately 80% of participants in both groups were transfused during the pre-Alpha/Alpha wave. Finally, 12.8% (n = 69) in the CCP and 13.2% (n = 70) in the control group were fully vaccinated at the time of transfusion (*P* = .94) (Table 1). The median number of symptoms at baseline for each group was 6 (IQR, 4–8) (*P* = .40), and the median time between symptom onset and transfusion for each group was 6 (4–7) days (*P* = .52).

**Table 1.** Characteristics of Trial Participants

Characteristic	Participants, No. (%)		*P* Value
	CCP Group (n = 538)	Control Group (n = 532)	
Age group			
ȃ18–34 y	159 (29.6)	150 (28.2)	.90
ȃ35–49 y	190 (35.3)	185 (34.8)	
ȃ50–64 y	151 (28.1)	160 (30.1)	
ȃ≥65 y	38 (7.1)	37 (7.0)	
Female sex	291 (54.1)	318 (59.8)	.07
Race			
ȃWhite	422 (78.4)	436 (82.0)	.17
ȃBlack	80 (14.9)	63 (11.8)	
ȃAsian	22 (4.1)	22 (4.1)	
ȃNative American	9 (1.7)	11 (2.1)	
ȃPacific Islander	1 (0.2)	2 (0.4)	
ȃOther	5 (0.9)	3 (0.6)	
BMI^a^			
ȃ<18	7 (1.3)	7 (1.3)	.51
ȃ18–25	159 (29.6)	135 (25.4)	
ȃ25–29	183 (34.0)	175 (32.9)	
ȃ30–34	105 (19.5)	117 (22.0)	
ȃ35–39	52 (9.7)	56 (10.5)	
ȃ≥40	32 (5.9)	42 (7.9)	
Timing of presentation			
ȃPre-Alpha/Alpha wave	435 (80.9)	431 (81.0)	>.99
ȃDelta wave	103 (19.1)	101 (19.0)	>.99
Baseline symptoms			
ȃFatigue	455 (84.6)	455 (85.5)	.94
ȃCough	422 (79.3)	422 (79.3)	.86
ȃRunny/stuffy nose	330 (61.3)	345 (64.8)	.27
ȃLoss of smell	283 (52.6)	299 (56.2)	.37
ȃHeadache	308 (57.2)	298 (56.0)	.50
ȃMyalgia	287 (53.3)	289 (54.3)	>.99
ȃLoss of taste	260 (48.3)	276 (51.9)	.48
ȃShortness of breath	194 (36.1)	197 (37.0)	.92
ȃSore throat	187 (34.8)	189 (35.5)	.97
ȃChills	144 (26.8)	168 (31.6)	.12
ȃDiarrhea	130 (24.2)	127 (23.9)	.89
ȃNausea/vomiting	131 (24.3)	109 (20.5)	.14
ȃNeurologic symptoms	74 (13.8)	75 (14.1)	>.99
ȃFever	80 (14.9)	73 (13.7)	.60
ȃSkin manifestations	21 (3.9)	22 (4.1)	>.99
ȃFull COVID-19 vaccination	69 (12.8)	70 (13.2)	.94
ȃReceipt of monoclonal antibodies	1 (0.2)	4 (0.8)	.34
Comorbid conditions			
ȃHypertension	130 (24.2)	120 (22.6)	.58
ȃAsthma	51 (9.5)	68 (12.8)	.15
ȃDepression	45 (8.4)	53 (10.0)	.55
ȃDiabetes	45 (8.4)	44 (8.3)	>.99
ȃAnxiety	37 (6.9)	32 (6.0)	.53
ȃCancer	27 (5.0)	23 (4.3)	.59
ȃVitamin D deficiency	9 (1.7)	20 (3.8)	.07
ȃCurrent smoker	31 (5.8)	19 (3.6)	.09
ȃHIV	12 (2.2)	11 (2.1)	.94
ȃArthritis	6 (1.1)	10 (1.9)	.48
ȃStroke	6 (1.1)	6 (1.1)	>.99
ȃFibromyalgia	3 (0.6)	5 (0.9)	.75
ȃLiver disorder	1 (0.2)	3 (0.6)	.64
ȃImmunosuppression	0 (0)	2 (0.4)	.47
ȃSolid organ transplant	2 (0.4)	1 (0.2)	.98
ȃPulmonary fibrosis	1 (0.2)	1 (0.2)	>.99

Abbreviations: BMI, body mass index; CCP, COVID-19 convalescent plasma; COVID-19, coronavirus disease 2019; HIV, human immunodeficiency virus.

BMI calculated as weight in kilograms divided by height in meters squared.

### Symptoms and Clusters at Day 14

On day 14 of follow-up, >70% of participants reported ≥1 symptom. Participants in the control and CCP groups each had a median of 2 symptoms (IQR, 0–4) (*P* > .99). The greatest proportion of participants in both groups experienced cough (CCP vs control group, 210 [39.0%] vs 221 participants [41.5%]; *P* = .37), followed by fatigue (207 [38.5%] vs 215 [40.4%]; *P* = .48), loss of smell (176 [32.7%] vs 183 [34.4%]; *P* = .53), and loss of taste (150 [27.9%] vs 146 [27.4%]; *P* > .99). Overall, there were no significant differences in the prevalence of each symptom at day 14 between the treatment groups. We also assessed the prevalence of clusters 1–3 at day 14. Cluster 1 (any cough, fatigue, shortness of breath, or headache) was the most common in both treatment groups (CCP group, 308 participants [57.2%]; control group, 325 participants [61.1%]; *P* = .16) (Table 2).

**Table 2.** Prevalence of Coronavirus Disease 2019 Symptoms and Clusters on Day 14

COVID-19 Symptoms	Participants, No. (%)		*P* Value
	CCP Group (n = 538)	Control Group (n = 532)	
Symptomatic with >1 symptom	381 (71.6)	381 (70.8)	.78
Symptom			
ȃCough	210 (39.0)	221 (41.5)	.37
ȃFatigue	207 (38.5)	215 (40.4)	.48
ȃLoss of smell	176 (32.7)	183 (34.4)	.53
ȃLoss of taste	150 (27.9)	146 (27.4)	>.99
ȃShortness of breath	91 (16.9)	111 (20.9)	.10
ȃRunny or stuffy nose	112 (20.8)	99 (18.6)	.42
ȃHeadache	80 (14.9)	96 (18.0)	.17
ȃ Myalgia	52 (9.7)	50 (9.4)	.99
ȃNeurologic symptoms	39 (7.2)	42 (7.9)	.75
ȃNausea/vomiting	22 (4.1)	28 (5.3)	.43
ȃSore throat	27 (5.0)	26 (4.9)	>.99
ȃDiarrhea	22 (4.1)	25 (4.7)	.71
ȃSkin manifestations	11 (2.0)	19 (3.6)	.17
ȃChills	10 (1.9)	15 (2.8)	.39
ȃFever	0 (0.0)	3 (0.6)	.24
Symptom cluster^a^			
ȃ1	308 (57.2)	325 (61.1)	.16
ȃ2	207 (38.5)	211 (39.7)	.68
ȃ3	129 (24.0)	154 (28.9)	.08

Abbreviations: CCP, COVID-19 convalescent plasma; COVID-19, coronavirus disease 2019.

Cluster 1 included fatigue, shortness of breath, headache, and/or cough; cluster 2, neurologic symptoms, loss of smell, and/or loss of taste; and cluster 3 (affecting the ability to return to work), any fever, any shortness of breath, grade 2+ fatigue, any diarrhea, grade 2+ headache, grade 2+ myalgia, and/or any vomiting.

Using the silhouette method, we identified 2 clusters as the optimal number of clusters to minimize intracluster variation for the k-means cluster analysis (Supplementary Figure 1). The first k-means cluster (K1) consisted of chills, diarrhea, fever, myalgia, nausea, vomiting, neurologic changes, shortness of breath, skin manifestations, and sore throat. The second cluster (K2) contained cough, fatigue, headache, runny/stuffy nose, loss of smell, and loss of taste. Full results of the k-means cluster derivation can be found in Supplementary Table 2. Of these 2 clusters, K2 had the highest proportion of participants experiencing those symptoms at day 14 (CCP vs control group, 369 [68.6%] vs 367 participants [69.0%]; *P* > .99) (Supplementary Table 3).

**Figure 1.**
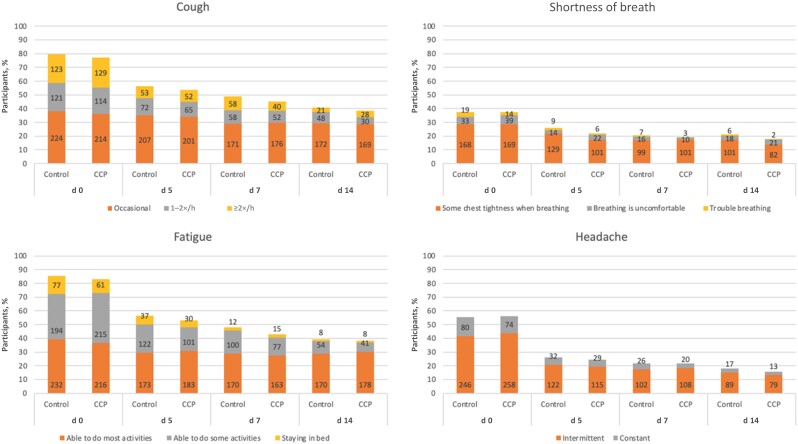
Severity of cluster 1 symptoms in both treatment groups at days 0, 5, 7, and 14. Numbers within bars indicate the number of participants experiencing a symptom, by severity level. Abbreviation: CCP, coronavirus disease 2019 convalescent plasma.

The severity of all cluster 1 symptoms decreased from day 0 to day 14 for participants in both groups. There were no significant differences between treatment groups in the change in severity from day 0 to day 14 for cough (*P* = .22), shortness of breath (*P* = .39), fatigue (*P* = .43), or headache (*P* = .44) (Figure 1). Moreover, in the subgroup of those reporting the highest severity of cluster 1 symptoms, there were no significant differences between treatment groups in the change in severity for cough (*P* = .51), shortness of breath (*P* = .99), or fatigue (*P* = .12), using ordinal regression analysis. There was an observed decrease in severity of headache for those in the CCP compared with the control group (odds ratio, 0.41; *P* = .02).

### Association Between Treatment and Time to Resolution of Symptoms by Day 14

By day 14, a total of 381 participants (71.6%) in the control group and 381 (70.8%) in the CCP group still had symptoms (*P* = .78). In the crude analysis, there was no significant difference in the subdistribution hazards of resolution of symptoms between treatment groups (subdistribution hazard ratio [_SD_HR], 0.99; *P* = .73). After adjustment for age, sex, race, BMI, diabetes mellitus, time from symptom onset to transfusion, vaccination status, C-reactive protein, and variant wave, the _SD_HR remained the same (*P* = .62) (Table 3). In subgroup analyses of the time to resolution of symptoms, there were no differences between treatment groups among male participants (_SD_HR, 0.94; *P* = .35), participants with diabetes mellitus at baseline (0.81; *P* = .21), or participants transfused within 5 days after symptom onset (0.91; *P* = .34) (Supplementary Table 4).

**Table 3.** Time to Resolution of Symptoms by Day 14

Analysis	Participants, No.		Events, No.	_SD_HR (CCP vs Control)	*P* Value
	CCP Group	Control Group			
Unadjusted	538	532	749	0.99	.73
Adjusted^a^	538	532	749	0.99	.62

Abbreviations: CCP, coronavirus disease 2019 convalescent plasma; _SD_HR, subdistribution hazard ratio.

Adjusted for age, sex, race, body mass index, vaccination status, diabetes, variant wave, C-reactive protein level, and time from symptom onset to transfusion.

### Sensitivity Analyses

Our results were robust and did not change when we excluded individuals with symptom resolution before day 3 (_SD_HR, 1.00; *P* = .93), vaccinated individuals (0.96; *P* = .42), or hospitalized participants (1.00; *P* = .98) (Supplementary Table 5).

## DISCUSSION

This analysis of clinical trial data from CSSC-004, investigating the impact of CCP for the treatment of COVID-19 on resolution of symptoms, has several important findings. In both the CCP and control treatment arms, 70% of participants still experienced COVID-19 symptoms at day 14. There was no statistically significant difference in the time to resolution of all symptoms by day 14 between CCP and control plasma. After clustering symptoms both on clinical significance and algorithmically, based on how they presented in the CSSC-004 study population, there were no significant differences in the prevalence of symptoms between treatment groups at day 14. Finally, looking at the prevalence of individual symptoms and the prevalence of any symptom at day 14, there were no significant differences between treatment groups.

As expected, the severity of symptoms decreased with time, even for those with the most advanced symptoms. Consistent with findings of other studies [16–18], the most common persistent symptoms were cough, fatigue, and loss of smell and taste; however, symptoms limiting the return to work, including fever, diarrhea, and vomiting, were far less common. This suggests that while patients may still have symptoms at 14 days, those symptoms may not affect their ability to return to the workforce.

Our results contribute to existing data on symptom resolution with the use of antibodies [19], and specifically CCP for the treatment of COVID-19 [20, 21]. Our results are in contrast to the benefit of CCP treatment found in the CSSC-004 trial, where there was decreased hospitalization and death with CCP treatment [14]. These results open the possibility that the timing of antibody treatment may affect the biologic pathway from infection to hospitalization and death differently from the pathway from infection to symptom progression. Further research is needed to understand the biologic mechanisms underlying the effects of treatment on the prevention of severe disease, compared with effects on symptom duration and resolution.

Therapy with monoclonal antibodies and CCP has been shown to reduce hospitalization and death rates when used early in the course of SARS-CoV-2 infection [14, 22]. Some studies of monoclonal antibodies have demonstrated an improvement in the time to resolution of symptoms [23, 19]. However, in our study there was no significant effect of early CCP treatment on resolution of any individual symptom or clusters of symptoms at 2 weeks, although the prevalence of most symptoms was slightly lower at day 14 in the CCP cohort.

This study had several notable strengths. First, the CSSC-004 study population was large and outpatient at enrollment, and the study was double masked. Second, high-titer CCP was used, in line with the current US Food and Drug Admsinistration's emergency use authorization [24]. Finally, data on symptoms were prospectively and directly obtained from participants through phone visits multiple times before day 10 and again at an in-person study visit on day 14. In addition to the presence or absence of symptoms, we collected data on the severity of those symptoms and were able to assess changes in severity over time. Study data on symptoms were collected uniformly across all sites, with a population representing broad demographics, a variety of patient populations, and 2 COVID-19 variants, thus increasing the generalizability of our findings.

Our analyses had limitations. First, the k-means cluster analysis enabled us to group symptoms based on their prevalence over the course of the disease. However, the biologic plausibility of the relationships within these symptom groupings was difficult to ascertain. This was possibly affected by the addition of symptoms to the data collection instruments as the clinical definition of COVID-19 evolved. Owing to masking of participants, differential reporting of symptoms is unlikely. Second, this study was conducted early in the pandemic, when relatively few participants were fully vaccinated. Results may differ for patients who have immunity from prior infection or vaccination. Finally, while we did not identify statistically significant differences in treatment effect between participants presenting in the Alpha and Delta waves, it is undetermined whether this will remain true for subsequent variants of concern.

As rates of hospitalizations and death after infection with SARS-CoV-2 have decreased, the focus has shifted, and new treatment trials are using symptom resolution as their outcome [23]. For future studies, standardization of symptom data collection and analysis will be important. Furthermore, understanding treatment effect in light of variability in symptom presentation between viral variants will be critical in determining patient quality of life and the ability to return to work. Different symptoms have different levels of impact on these 2 criteria. It is undetermined whether or not improvement in specific symptoms is sufficient for a treatment to be clinically meaningful. Our results highlight the need for future trials to assess symptoms in the acute illness phase to understand the impact on patients’ ability to return to work.

## Supplementary Data

Supplementary materials are available at *The Journal of Infectious Diseases* online. Consisting of data provided by the authors to benefit the reader, the posted materials are not copyedited and are the sole responsibility of the authors, so questions or comments should be addressed to the corresponding author.

## Supplementary Material

jiad023_Supplementary_Data

## Notes

***Acknowledgments.*** The authors gratefully acknowledge the plasma donors and study participants who generously gave of their time and provided biologic specimens and the passionate staff who participated in the CSSC-001 and CSSC-004 trials.

***Disclaimer.*** The study sponsors did not contribute to the study design, the collection, analysis, and interpretation of data, or the decision to submit the manuscript for publication.

***Financial support.*** This work was supported by the US Department of Defense's Joint Program Executive Office for Chemical, Biological, Radiological and Nuclear Defense, in collaboration with the Defense Health Agency (contract W911QY2090012), as well as by Bloomberg Philanthropies; the State of Maryland; the National Institute of Allergy and Infectious Diseases (NIAID) (grant 3R01AI152078-01S1), the National Center for Advancing Translational Sciences (grant U24TR001609), and the Division of Intramural Research, NIAID, National Institutes of Health; the Mental Wellness Foundation; the Moriah Fund; the Octapharma; the HealthNetwork Foundation, and the Shear Family Foundation. Funding to pay the Open Access publication charges for this article was provided by National Institutes of Health.
