# Supplementary material for: Symptom Duration and Resolution With Early Outpatient Treatment of Convalescent Plasma for Coronavirus Disease 2019: A Randomized Trial
Source: J Infect Dis. 2023 Jan 31;227(11):1266–73. doi: 10.1093/infdis/jiad023 (PMC10226658; doi:10.1093/infdis/jiad023)
Supplement: jiad023_Supplementary_Data [file jiad023_supplementary_data.zip › Supplemental_Table_1.docx]

| **Supplemental Table 1. Severity grades for each collected COVID-19 symptom*** | | | |
| --- | --- | --- | --- |
| **Symptom** | **Grade 1** | **Grade 2** | **Grade 3** |
| Cough | Occasional | 1-2x per hour | 2+ per hour |
| Fatigue | Able to do most activities | Able to do some activities | Staying in bed |
| Loss of smell | Able to smell most things normally | Able to smell just a few things normally | Unable to smell anything |
| Loss of taste | Able to taste most things normally | Able to taste just a few things normally | Unable to taste anything |
| Shortness of breath | Some chest tightness when breathing | Breathing is uncomfortable | Trouble breathing |
| Stuffy nose | Intermittent | Constant | -- |
| Runny nose | Occasional | 1-2x per hour | 2+ per hour |
| Headache | Intermittent | Constant | -- |
| Myalgia | Present in past 24 hours, but no hampered movement | Hampers movement | -- |
| Neurological symptoms | Changes present | -- | -- |
| Nausea | Intermittent | Constant | -- |
| Vomiting | 1-2x per day | 3-4x per day | 5(+)x per day |
| Sore throat | Present in past 24 hours, no difficulty swallowing | Present in past 24 hours, difficulty swallowing | -- |
| Diarrhea | 2 times as many as normal bowel movements in a day | 3 or more times as many as normal bowel movements in a day | Loose bowel movements |
| Skin manifestations | Present | -- | -- |
| Chills | Present in past 24 hours | -- | -- |
| Fever | Fever (>100.5 degrees F) in past 24 hours | Feverish in past 24 hours | -- |

* All symptom data collection forms allowed for the recording of “not present” for each symptom. Definitions of each grade of each symptom was determined by investigator consensus during the planning of the trial.
