# Supplementary material for: Symptom Duration and Resolution With Early Outpatient Treatment of Convalescent Plasma for Coronavirus Disease 2019: A Randomized Trial
Source: J Infect Dis. 2023 Jan 31;227(11):1266–73. doi: 10.1093/infdis/jiad023 (PMC10226658; doi:10.1093/infdis/jiad023)
Supplement: jiad023_Supplementary_Data [file jiad023_supplementary_data.zip › Supplemental_Table_2.docx]

| **Supplemental Table 2. Derivation of clusters using k-means** | | | | | |
| --- | --- | --- | --- | --- | --- |
| 2 cluster pre-specification | | 3 cluster pre-specification | | 4 cluster pre-specification | |
| Cluster | Symptom | Cluster | Symptom | Cluster | Symptom |
| 1 | chills | 1 | chills | 1 | cough |
|  | diarrhea |  | cough |  | fatigue |
|  | fever |  | fatigue | 2 | chills |
|  | myalgia | 2 | diarrhea |  | diarrhea |
|  | nausea/vomiting |  | fever |  | fever |
|  | neurological changes |  | nausea/vomiting |  | nausea/vomiting |
|  | shortness of breath |  | neurological changes |  | neurological changes |
|  | skin manifestations |  | loss of smell |  | skin manifestations |
|  | sore throat |  | skin manifestations |  | sore throat |
| 2 | cough |  | sore throat | 3 | loss of smell |
|  | fatigue | 3 | headache |  | loss of taste |
|  | headache |  | myalgia | 4 | headache |
|  | stuffy/runny nose |  | stuffy/runny nose |  | myalgia |
|  | loss of smell |  | shortness of breath |  | stuffy/runny nose |
|  | loss of taste |  | loss of taste |  | shortness of breath |
