# Supplementary material for: Symptom Duration and Resolution With Early Outpatient Treatment of Convalescent Plasma for Coronavirus Disease 2019: A Randomized Trial
Source: J Infect Dis. 2023 Jan 31;227(11):1266–73. doi: 10.1093/infdis/jiad023 (PMC10226658; doi:10.1093/infdis/jiad023)
Supplement: jiad023_Supplementary_Data [file jiad023_supplementary_data.zip › Supplemental_Table_3.docx]

| **Supplemental Table 3. Prevalence of algorithmic clusters at Day 14** | | | |
| --- | --- | --- | --- |
|  | **CCP** | **Control** | **P-Value** |
|  | **(N=538)** | **(N=532)** |  |
| **Cluster*** |  |  |  |
| K1 | 172 (32.0%) | 188 (35.3%) | 0.32 |
| K2 | 369 (68.6%) | 367 (69.0%) | 1.00 |
| *K1: chills, diarrhea, fever, myalgia, nausea, vomiting, neurological changes, shortness of breath, skin manifestations, and sore throat;  K2: cough, fatigue, headache, runny/stuffy nose, loss of smell, and loss of taste | | | |
