# Supplementary material for: Symptom Duration and Resolution With Early Outpatient Treatment of Convalescent Plasma for Coronavirus Disease 2019: A Randomized Trial
Source: J Infect Dis. 2023 Jan 31;227(11):1266–73. doi: 10.1093/infdis/jiad023 (PMC10226658; doi:10.1093/infdis/jiad023)
Supplement: jiad023_Supplementary_Data [file jiad023_supplementary_data.zip › Supplemental_Table_4.docx]

| **Supplemental Table 4. Time to resolution of all symptoms by day 14 for subgroups** | | |
| --- | --- | --- |
|  | **Sub-distribution Hazard Ratio (CCP vs. Control Plasma)** | **P-Value** |
| **Sex** |  |  |
| Male | 0.935 | 0.35 |
| Female | 1.015 | 0.80 |
| **Age** |  |  |
| 18-34 | 1.097 | 0.31 |
| 35-49 | 0.978 | 0.77 |
| 50-64 | 0.887 | 0.14 |
| 65+ | 1.044 | 0.83 |
| **White** | 0.987 | 0.79 |
| **Hispanic/Latino** | 1.180 | 0.19 |
| **BMI** |  |  |
| <18* | 14.871 | <0.001 |
| 18-25 | 1.097 | 0.34 |
| 25-29 | 0.913 | 0.23 |
| 30-34 | 1.097 | 0.30 |
| 35-39 | 0.867 | 0.32 |
| 40+ | 0.777 | 0.21 |
| **Diabetes** |  |  |
| Yes | 0.811 | 0.21 |
| No | 0.994 | 1.09 |
| **Transfusion under 5 days from symptom onset** | 0.911 | 0.34 |
| **Presentation during pre-Alpha/Alpha wave** | 0.984 | 0.74 |
| **Presentation during Delta wave** | 0.939 | 0.58 |
| * N=14 | | |
