# Supplementary material for: Symptom Duration and Resolution With Early Outpatient Treatment of Convalescent Plasma for Coronavirus Disease 2019: A Randomized Trial
Source: J Infect Dis. 2023 Jan 31;227(11):1266–73. doi: 10.1093/infdis/jiad023 (PMC10226658; doi:10.1093/infdis/jiad023)
Supplement: jiad023_Supplementary_Data [file jiad023_supplementary_data.zip › Supplemental_Table_5.docx]

| **Supplemental Table 5. Sensitivity analyses for the association between CCP and resolution of symptoms** | | | | | |  |
| --- | --- | --- | --- | --- | --- | --- |
|  | **CCP** | **Control** | **Events** | **Sub-distribution Hazard Ratio (CCP v. Control)** | **P-Value** | |
| **3-day lag on resolution, adjusted*** | 538 | 532 | 749 | 1.001 | 0.93 | |
| **Unvaccinated only, adjusted** | 469 | 462 | 657 | 0.962 | 0.42 | |
| **Excluding hospitalized, adjusted**** | 524 | 503 | 718 | 1.002 | 0.98 | |
| * Resolution prior to Day 3 was not counted as an event for this analysis | | | | | |  |
| ** Excluding those who were hospitalized/died during trial follow-up from this analysis | | | | | |  |
