# Supplementary figures and images for: Symptom Duration and Resolution With Early Outpatient Treatment of Convalescent Plasma for Coronavirus Disease 2019: A Randomized Trial
Source: J Infect Dis. 2023 Jan 31;227(11):1266–73. doi: 10.1093/infdis/jiad023 (PMC10226658; doi:10.1093/infdis/jiad023)

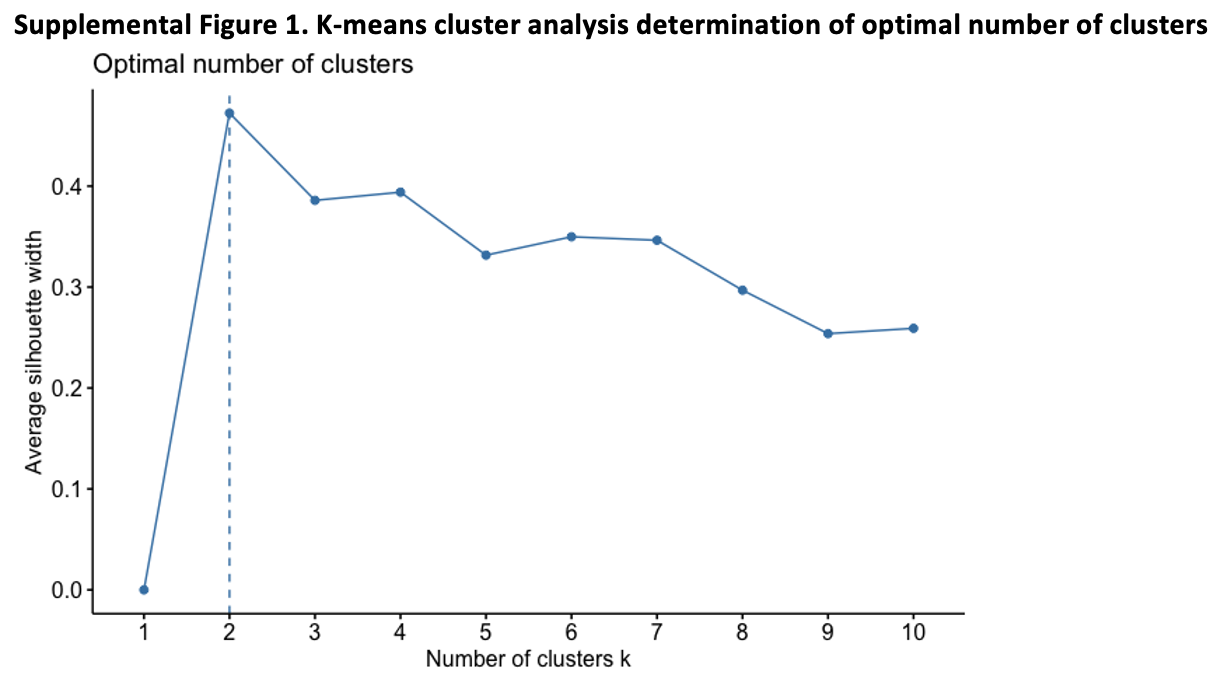

Supplement: jiad023_Supplementary_Data [file jiad023_supplementary_data.zip › supplemental_figure1.png]
